# Baseline Prognostic Factors and Statistic Model to Predict Early Virological Response in Telbivudine-Treated Patients With Chronic Hepatitis B

**DOI:** 10.5812/hepatmon.15573

**Published:** 2013-12-23

**Authors:** Rui Zhou, Yuan-Ping Zhou, Chun Lin, Hai-bing Gao, Shui-wen Huang, Zu-xiong Huang, Fang Sun, Yong Lin, Dong-qing Zhang, Qing-feng Lin, Wen Ao, Chen Pan

**Affiliations:** 1 Department of Infectious Diseases, Nanfang Hospital, Southern Medical University, Guangzhou, China; 2 Infectious Disease Hospital, Meng Chao Hepatobiliary Hospital, Fujian Medical University, Fuzhou, China

**Keywords:** Hepatitis B, Chronic, Telbivudine, Proportional Hazards Models, Virology

## Abstract

**Background:**

Hepatitis B virus (HBV) infection is still a worldwide disease, which may cause liver cirrhosis or even hepatocellular carcinoma. Telbivudine is a potent nucleoside analogue used in the treatment of chronic hepatitis B (CHB); however, drug resistance has remained a challenge. As early virological response can predict long-term efficacy of nucleotide analogue treatment, numerous studies have been conducted in this area.

**Objectives:**

The aim of this study was to establish baseline prognostic factors and a statistical model to predict early virological response in telbivudine-treated CHB patients.

**Patients and Methods:**

One hundred and eight CHB patients without any experience of nucleotide analogue therapy were assigned to receive telbivudine (600 mg, once daily) for at least 24 weeks, and then were followed up every two weeks. Cox proportional hazard regression model analyses were employed to evaluate baseline variables, and further developing a statistical model to predict early virological response.

**Results:**

Negative family history of HBV infection (P = 0.000235), baseline higher serum TBIL (P = 0.038714) and AST (P = 0.020684) concentrations, and lower level of HBV-DNA (P = 0.0034784) were identified to be associated with higher possibility of early virological response. A model was established based on these variables to calculate the risk scores (R) for CHB patients. R > -0.38 suggested early virological response to telbivudine. The model was validated among an independent set of 20 patients.

**Conclusions:**

Family history as well as baseline bilirubin, AST and HBV DNA levels can predict early virological response. The model provides a better tool for response prediction based on the four prognostic factors.

## 1. Background

Chronic hepatitis B virus (HBV) infection is a major health problem (1-3). About 400 million people live with chronic hepatitis B virus (HBV) infection, and the risk of progression to end-stage complications such as cirrhosis and hepatocellular carcinoma has been correlated with persistent HBV replication, as reflected by serum HBV DNA levels (4). Correspondingly, prolonged suppression of HBV replication with antiviral therapy has been linked to reduced risks of end-stage disease manifestations, a finding that underscores the importance of long-lasting HBV suppression as a primary treatment goal (5-12). 

Telbivudine is an orally bioavailable L-nucleoside with potent and specific anti-HBV activity (13). Initial clinical trials had indicated that this agent induced a greater reduction in serum HBV DNA level, and less drug-resistance occurred compared to lamivudine (14, 15). 

Telbivudine had no mutagenic or carcinogenic effects, and no appreciable embryonic or fetal toxic effects, particularly important for men and women in their reproductive ages, according to many preclinical safety studies of the agent (16). A meta-analysis also demonstrated that telbivudine application in late pregnancy is effective in the interruption of intrauterine HBV infection, with no significant adverse effects or complications (17). 

Having so many distinct advantages, telbivudine has been widely used in clinical practice. Nonetheless, development of resistance mutations in the HBV polymerase-reverse transcriptase has limited its application. Therefore, it is very important to identify those who benefit most from this therapy.

## 2. Objectives

Studies have shown that early virological response predicts the long-term outcomes of nucleotide analogue treatment for patients with chronic HBV infection (18-20). However, it takes several weeks or even months waiting for the result of the response. If the patient failed to respond to the drug, it would be a waste of time and money, and causes delay in appropriate antiviral therapy. Therefore, we studied CHB patients’ baseline characteristics to establish prognostic factors and a statistical model to predict early virological response reflecting the long-term effect of telbivudine.

## 3. Patients and Methods

### 3.1. Ethics

The present study was an observational study, and ethical issues were considered during the data collection and preservation. The Ethics Committee of Infectious Disease Hospital, MengChao Hepatobiliary Hospital, Fujian Medical University approved the study; all patients gave an informed consent. 

### 3.2. Patients

One hundred and eight telbivudine-treated CHB patients (age, 16-65 years) in Infectious Disease Hospital, MengChao Hepatobiliary Hospital, Fujian Medical University from January 2007 until April 2012 were recruited. Enrolled cases included some HBeAg-negative ones. All patients met the following diagnostic criteria for CHB, and had indications for telbivudine treatment:

Patients with positive results for serum HBsAg for longer than six months; serum alanine aminotransferase (ALT) level, 2 to 10times of the upper limit of normal (ULN); or serum HBV DNA level more than 104 IU/mL.

### 3.3. Exclusion Criteria

Exclusion criteria were as follows; coinfection with hepatitis C, hepatitis D, or the human immunodeficiency virus (HIV); evidence of hepatic decompensation, pancreatitis, or hepatocellular carcinoma; previous treatment for hepatitis B with nucleoside or nucleotide analogues or both; treatment with interferon or other immunomodulators within the previous 12 months; Other forms of liver disease， such as NAFLD, alcoholic hepatitis, and drug induced hepatitis; serum creatinine level > 1.5 mg per deciliter (133 μmol per liter); serum amylase or lipase level > 1.5 times the upper limit of normal; prothrombin time more than > 3 seconds; serum albumin level < 33 g/L; and bilirubin level > 2.0 mg/dL (50 μmol/L). Patients with serum alpha fetoprotein (AFP) levels > 50 ng/mL were enrolled only in case of excluding underlying hepatocellular carcinoma. 

### 3.4. Study Design

Eligible subjects were assigned to receive telbivudine therapy (600 mg, q.d., PO) for at least 24 weeks. Primary data analyses were specified for a 24-week treatment period.

Patient histories were obtained, physical examinations were conducted, and laboratory assessments were performed at baseline, and weeks 2, 4, 6, 8, 10, 12, 14, 16, 18, 20, 22, and 24. Serum albumin, globulin, total bilirubin, direct/indirect bilirubin, alanine aminotransferase (ALT), aspartate aminotransferase (AST), glutamyltranspeptidase (GGT), HbeAg, creatinine, amylase, lipase, and prothrombin time were tested by standardized methods in Infectious Disease Hospital, MengChao Hepatobiliary Hospital, Fujian Medical University Clinical Laboratory. ABI 7500 Real-Time PCR was used to quantitatively detect serum HBV DNA levels using HBV PCR kit (FuXing, Shanghai, China); the detection limit was 420 IU/mL. 

### 3.5. Definition of Early Virological Response 

Early virological response was defined as suppression of plasma HBV DNA to less than 420 IU/mL at week 24 according to the PCR analysis.

### 3.6. Statistical Analysis 

In our study all telbivudine-treated patients were considered for the analyses. Enrolled patients were randomly assigned into two groups by using a table of random number with an allocation ratio of 85%:15%. Eighty five percent of patients were studied to determine predictors and establish a statistical model, and the rest for the model validation. 

The demographics of patients in the model group and model validation group were compared by Student’s t test for continuous data, and x^2^ test for dichotomous variables. Cox proportional-hazards regression was adopted to screen predictors from the baseline parameters and develop a model for early virological response. Receiver operating characteristic (ROC) curve and area under curve (AUC) were applied to assess the optimal cutoff values of the model through maximizing the Youden’s index. 

A P value < 0.05 was considered statistically significant. A 95% confidence interval accompanied all estimates when it was appropriate. All the analyses were performed by SPSS version 13.0 (SPSS Inc., IL, The USA). 

## 4. Results

### 4.1. Demographic Characteristics and Baseline Parameters

One hundred and eight patients were recruited. Table 1 presents descriptive statistics on patient features recorded at baseline. No patient withdrew from the study before the 24th week. At 24th week, 69.4% of patients showed suppression of plasma HBV DNA level to < 420 IU/mL. 

**Table 1.** Baseline Demographic, Clinical, Biochemical and Virological Features

Variables	Model (n = 88)^a^	Validation (n = 20)^b^	P value
**Demographic, mean ± SD**			
Age, y	31.0 ± 9.4	30.0 ± 10	0.674
**Gender, %**			0.945
Man	80.7	80	
Women	19.3	20	
**Clinical Family History, %**			0.693
Yes	60.2	65	
No	39.8	35	
**History of HBV Infection, mean ± SD, y**	8.0 ± 7.6	11.5 ± 8.8	0.246
**Biochemical, mean ± SD**			
Alb^c^, g/L	42.0 ± 4.7	41.0 ± 8.2	0.072
Glb ^c^, g/L	31.0 ± 6.7	33.0 ± 3.5	0.085
TBIL ^c^, μmol/L	17.8 ± 6.8	16.6 ± 6.7	0.485
IBIL ^c^, μmol/L	13.8 ± 22.3	12.6 ± 4.4	0.434
ALT ^c^, U/L	122.0 ± 126.6	161.5 ± 72.5	0.742
AST ^c^, U/L	80.50 ± 75.4	114.0 ± 52.5	0.464
GGT ^c^, U/L	52.5 ± 43.7	74.5 ± 47.5	0.290
**Virological, mean ± SD**			
log 10, HBVDNA, IU/mL	7.3 ± 1.1	7.12 ± 0.7	0.751
**HBeAg, %**			0.721
positive	87.5	85	
negative	12.5	15	

^a^ Patients studied to establish prognostic factors and statistical model to predict early virological response.

^b^ Patients studied to validate the model.

^c^ Abbreviations: SD, standard deviation; Alb, albumin; Glb, globulin; TBIL, total bilirubin levels; DBIL, direct bilirubin; ALT, alanine aminotransferase; AST, aspartate aminotransferase; GGT, gamma-glutamyl transpeptidase; HBeAg, hepatitis B e-antigen.

### 4.2. Predictors of Early Virological Response 

Eighty-eight patients were studied to determine predictors of early virological response and to develop a prediction model. Cox Proportional Hazard Regression Model analyses were employed to evaluate baseline variables, including gender, age, family history of HBV infection, history of HBV infection, serum concentrations of ALB,GLB,TBIL,IBIL, ALT,AST, GGT, HBV DNA level, and HBeAg. 

The results showed that family history negative patients had a 3.18 time (P = 0.0002235) greater chance of early virological response. Patients with higher serum concentrations of TBIL (P = 0.038714) and AST (P = 0.020684) were more likely to achieve early virological response. On the contrary, lower level of HBV DNA (P = 0.0034784) suggested higher possibility of early virological response. Other factors had no predictive value (Table 2). 

**Table 2.** Variables Assessed Among 88 Patients to Establish Prognostic Factors and Statistical Model for Early Virological Response

Variables	B^a^	SE^b^	df	P value
**Age, y**	-0.0057	0.015531	1	0.713505
**Gender**	0.353132	0.40574	1	0.384114
**Family history**	1.157655	0.31477	1	0.000235
**History of HBV Infection**	0.020759	0.020994	1	0.322762
**Alb ** ^**c**^	-0.00626	0.033688	1	0.852678
**Glb ** ^**c**^	0.007749	0.023556	1	0.742183
**TBIL ** ^**c**^	0.05488	0.026548	1	0.038714
**IBIL ** ^**c**^	0.004015	0.006644	1	0.545639
**ALT ** ^**c**^	-0.00238	0.001369	1	0.082262
**AST ** ^**c**^	0.00586	0.002533	1	0.020684
**GGT ** ^**c**^	0.000651	0.003834	1	0.865192
**HbeAg ** ^**c**^	-0.63333	0.427528	1	0.138504
**log10** ** (HBV-DNA)**	-0.27186	0.128789	1	0.034784

^a^ Partial regression coefficient.

^b^ Standard error of partial regression coefficient.

^c^ Abbreviations: Alb, albumin; Glb, globulin; TBIL, total bilirubin levels; DBIL, direct bilirubin; ALT, alanine aminotransferase; AST, aspartate aminotransferase; GGT, gamma-glutamyl transpeptidase; HBeAg, hepatitis B e-antigen.

### 4.3. Early Virological Response Modeling 

Risk scores (R) for individual patients were estimated by combining the four prognostic variables with the Partial Regression Coefficient reported in Table 2. That is, R = 1.158*LN (family history of HBV infection, 1 = Yes, 2 = no) +0.055*LN (TBIL μmol/L) + 0.006*LN (AST U/L) - 0.272*LN (log10HBV-NDA IU/mL). For example, a hypothetical CHB patient with no family history and serum TBIL of 10.5μmol/L, serum AST of 258U/L, and HBV-DNA Level of 6.0E + 6IU/mL, the risk score would be calculated as follows: R = 1.158*LN (1) + 0.055*LN (10.5) + 0.006*LN (258) - 0.272*LN (log106.0E + 6) = - 0.378. 

### 4.4. Model Validation

Twenty independent patients were studied to validate the model accuracy. Their risk scores (R) were calculated according to the equation mentioned above. The receiver operating characteristic (ROC) curve was drawn by combining the risk scores and the actual occurrence of early virological response at week 24. Actually there were 15 patients achieved early virological response at that time point. The area under the curve (AUC) was 73.3%, with a 95% confidence interval (CI) of 50.2% to 104.4% (P = 0.044). The optimal cutoff was determined through maximizing the Youden’s index (J), J = sensitivity + specificity-1. The optimal cutoff value was -0.38403, with a sensitivity of 100% and a specificity of 60%.

## 5. Discussion

Telbivudine is a safety oral drug with potent and specific anti-HBV activity. Telbivudine treatment reduces risks of end-stage disease manifestations such as cirrhosis and hepatocellular carcinoma. However, its use has been limited due to the development of resistance mutations in the HBV polymerase-reverse transcriptase. It was reported that 5.0% and 2.3% of HBeAg-positive and HBeAg-negative patients developed resistance to the drug at week 52, and at week 104 the resistance rates were 17.8% and 7.3% (21, 22). Telbivudine-resistance is rather a big problem: additional drugs such as adefovir are required to suppress the virus, and the patients would have to bear an increased economic burden and higher risk of developing end-stage disease. If we predict the effect of telbivudine before administration, many medical resources would be saved, and many subsequent problems would be avoided. Therefore, we conducted this study. In this study, a model using serum concentrations of bilirubin and ALT, plasma HBV DNA level, and family history of HBV infection, accurately predicted the early virological response (R > -0.38). The model accuracy was validated in an independent patient group. As early virological response indicates the outcomes of nucleotide analogue treatment for chronic HBV infection, (18-20) , our model seems to be reliable to help telbivudine therapy decision making.

As a useful predictor of prognosis, early virological response has been adopted to guide clinical practice. During telbivudine treatment, for example, we can decide whether to continue the therapy, or to add on or switch to another drug according to the response. However, if no response or incomplete response occurs at week 24, it indicates that resistance mutations may have happened or is more likely to happen in the future, subsequent therapy would be quite difficult. On this situation, early virological response is not an ideal predictor for nucleotide analogue treatment.

It was reported that baseline ALT and HBV DNA predict the virological response of lamivudine treatment at weeks 52 and 156 (23). Another study showed that baseline HBV DNA levels < 9 logs copies/mL and ALT level ≥ 2 ×ULN are associated with better long-term outcome and lower risk of YMDD mutations compared to HBV DNA levels > 9 logs copies/mL or ALT level < 2 × ULN during lamivudine treatment for chronic hepatitis B (24). Similarly, baseline ALT and HBV DNA were found to be used as predicted values of other nucleoside analogues and interferon treatment. However, several defects existed in these studies. First, they did not use multivariate analysis to evaluate all candidate variables, hence the accuracy of the results was not guaranteed. Secondly, they did not combine the multivariate analysis with time as a variable to evaluate the predictor of outcomes. Thirdly, they did not provide a model to predict the outcomes of telbivudine therapy.

In a word, baseline ALT, baseline HBV DNA and early virological response are not perfect predictors in the prediction of telbivudine treatment effect in clinical practice.

As a main statistical tool for survival modeling, Cox proportional-hazards regression (25) was employed in the study to evaluate baseline variables and further developing a statistical model to predict early virological response. The candidate variables included gender, age, family history of HBV infection, history of HBV infection, and baseline serum concentrations of ALB, GLB, TBIL, IBIL, ALT, AST, GGT, HBV DNA level, and HBeAg. The early virological response time was defined as the time period from the beginning of telbivudine administration to the onset of the response. The term ‘‘censored’’ indicates the absence of early virological response at week 24 or lost to follow-up. We analyzed the association between early virological response and the candidate variables at baseline in all telbivudine recipients in our study to determine predictors of early virological response. Then the predictors and partial regression coefficient were combined to develop a model for response prediction, and risk scores (R) were calculated for validation of the model. The Receiver Operating Characteristic (ROC) curve was plotted by combining the risk scores and the actual occurrence of early virological response at week 24 actually. To validate the nomograms, we compared the actual occurrence of the response with the predicted response rates. Theoretically, an area under the curve (AUC) greater than 50% suggests the model utility, and greater than 70% indicates moderate utility. Further, we calculated standard indices of validity such as sensitivity, specificity, and AUC to determine the optimal cutoff. 

The results showed that negative family history (P = 0.000235), higher serum TBIL (P = 0.038714), and AST (P = 0.020684) concentrations and lower level of HBV-DNA (P = 0.0034784) were predictive values of early virological response.

Brook MG et al. studied 114 alpha-interferon treated CHB patients and found that loss of HBsAg in addition to HBeAg and hepatitis B virus DNA was more likely to occur in patients with chronic infection of less than two years duration (26). It supported the claim that newly acquired HBV infection is in association with better outcomes of antiviral therapy. Having a positive family history indicates that patients may be infected with HBV for a long time. On the contrary, patients without family history are more likely to have a shorter duration of infection. So family history may have a prognostic value, and was confirmed to be a predictor of early virological response in the present study. To our surprise, however age and history of HBV infection were not identified as predictive factors in our study, but many of the patients may have infected with HBV long before the diagnosis was made. Therefore, the actual significance of age and HBV infection history in prognosis of telbivudine-treated patients is still to be determined. 

It is known by liver biopsy that active CHB patients with higher level of viral replication are more likely to respond to alpha-interferon therapy. As serum concentrations of bilirubin and aspartate aminotransferase (AST) reflect the activity of chronic hepatitis B to a certain degree, it is easy to understand that the two factors were identified as predictors of the early virological response. However, alanine aminotransferase (ALT), which also reflects the grade of hepatitis activity on liver biopsy, was not found to be associated with early virological response in the present study. The possible explanation is that ALT is more susceptible than AST to be affected by some drugs. It was reported that CHB patients with lower level of HBV-DNA are more likely to respond to alpha-interferon and lamivudine therapy (27). Our study also demonstrated that lower baseline level of HBV DNA was associated with significantly higher possibility of early virological response.

Factors used to establish our model were baseline levels of bilirubin, AST, HBV-DNA and family history. All these are objective and relatively stable variables. By combining the four variables with the partial regression coefficient, we developed a model to predict early virological response. That is, R = 1.158*LN (family history of HBV infection, 1 = Yes, 2 = no) + 0.055*LN (TBIL μmol/L) + 0.006*LN (ASTU/l) - 0.272*LN (log10HBVNDA IU/mL). The model was validated in an independent set of patients. The area under the ROC curve (AUC) was 73.3% (P = 0.044), and the optimal cut off value was -0.38403. It means that patients with R > -0.38 are most likely to achieve early virological response to telbivudine therapy, or good outcomes of the treatment. Patients with R > -0.38 are most suitable for telbivudine treatment in clinical practice.

The model and optimal cutoff value were established from a short-term follow-up study of only telbivudine-treated CHB patients, which makes the conclusion a little weaker. Whether the model and cutoff value can be applied to other nucleos(t)ide analogs therapy for CHB needs to be determined. Furthermore, developing a more accurate model and cutoff values to predict the outcome of telbivudine treatment in CHB patients requires a large-scale, multicenter and long-term follow-up study. But, in the present study, the data were all from a single center, with a small sample size and a short follow-up period (only 24 weeks). Lastly, route of HBV transmission (Horizontal vs. vertical)，and the viral characteristics (genotype, HBeAg status) could affect the predictive model. However, as the exact route of HBC transmission for each patient is rather difficult to determine, and also for economic reasons, these factors were not included in the study. Therefore, whether the model and optimal cutoff value can accurately predict the early virological response and long-term outcome of telbivudine treatment should be further studied.
